# Cirrhotic patients in icu with gastro-intestinal bleeding managed according to recent guidelines display altered brain hemoglobin oxygen’s saturation assessed by near infrared spectroscopy

**DOI:** 10.1186/2197-425X-3-S1-A692

**Published:** 2015-10-01

**Authors:** D Thabut, M Mallet, S Tripon, M Rudler, N Weiss, Brain Liver Pitie-Salpetriere Study Group (BLIPS)

**Affiliations:** Assistance Publique - Hopitaux de Paris, La Pitié-Salpetriere Hospital, Hepatological ICU, Paris, France; Neurology Department, Assistance Publique - Hopitaux de Paris, La Pitié-Salpetriere Hospital, Neurological ICU, Paris, France

## Introduction

Near Infrared Spectroscopy (NIRS) is a non-invasive optical technique allowing a continuous measurement of brain's hemoglobin saturation in oxygen (rSO2). It is considered as a surrogate marker of cerebral insult, and recognized as a useful tool in in cardiovascular surgery and neuromonitoring. A rSO2< 50% is associated with increased neurological impairment and post-operative mortality. In cirrhotic patients with gastrointestinal bleeding (GIB), hemoglobin (Hb) threshold for transfusion has been recently lowered to 7 g/dL. Some patients develop hepatic encephalopathy (HE) after GIB. In subarachnoid hemorrhage, a threshold of 7 g/dL of Hb could worsen neurological outcome.

## Objectives

The aim of this study was to assess brain oxygenation using NIRS in cirrhotic patients with acute GIB admitted to ICU and managed according to recent guidelines, and to determine if brain injury was associated with Hb levels.

## Methods

Cirrhotic patients admitted in ICU for acute GIB were prospectively included. Bilateral continuous recording of rSO2 was started upon admission using a NIRS monitor (INVOS 5100c Cerebral Oxymeter (Covidien©) with two sensors placed on the patient's forehead. Minimal rSO2 (mini rSO2), average rSO2 (avr rSO2) and AUC of rSO2 50% (AUC50% rSO2), an integrated parameter depending on the depth/duration of desaturation under 50%, were extracted.

## Results

26 patients were included (median age: 60 years; 69%men). Etiology of cirrhosis was alcoholic 54%/ viral 19%/ NASH 23%/other 4%; Child Pugh A 15%/ B 20%/ C 65% and median MELD score 18. Median initial Hb was 7,9 g/dL and nadir within 24 first hours was 7,8g/dL. 14 patients (54%) had a nadir of Hb below 8 g/dL within the 24 first hours, and 15 (58%) patients were transfused. Median mini rSO2 was 37% right/37% left, avr rSO2 46% right/48% left and AUC50% rSO2 1138 right/698 left. 22 patients (85%) had mini rSO2< 50%. Mini rSO2 was significantly lower in patients having a nadir of Hb below 8g/dL. Mini rSO2, avr rSO2 and AUC50% rSO2 were independently correlated to initial Hb (p < 0.01 for all), nadir of Hb within the 24 first hours (p < 0.005 for all), and MELD score (p < 0.05 for all).

## Conclusions

85% of cirrhotic patients admitted to ICU for acute GIB and managed according to recent guidelines displayed mini rSO2 below 50% within 24 hours after admission. Low Hb levels within the 24 first hours were associated with brain desaturation. Further studies are mandatory to assess the influence of Hb thresholds on the development of HE.

